# Light pollution disrupts sleep in free-living animals

**DOI:** 10.1038/srep13557

**Published:** 2015-09-04

**Authors:** Thomas Raap, Rianne Pinxten, Marcel Eens

**Affiliations:** 1 Department of Biology, Ethology group, University of Antwerp, Universiteitsplein 1, B-2610 Wilrijk, Belgium; 2 Faculty of Social Sciences, Didactica research group, University of Antwerp, Prinsstraat 13, B-2000, Antwerp, Belgium

## Abstract

Artificial lighting can alter individual behaviour, with often drastic and potentially negative effects on biological rhythms, daily activity and reproduction. Whether this is caused by a disruption of sleep, an important widespread behaviour enabling animals to recover from daily stress, is unclear. We tested the hypothesis that light pollution disrupts sleep by recording individual sleep behaviour of great tits, *Parus major,* that were roosting in dark nest-boxes and were exposed to light-emitting diode light the following night. Their behaviour was compared to that of control birds sleeping in dark nest-boxes on both nights. Artificial lighting caused experimental birds to wake up earlier, sleep less (–5%) and spent less time in the nest-box as they left their nest-box earlier in the morning. Experimental birds did not enter the nest-box or fall asleep later than controls. Although individuals in lit nest-boxes did not wake up more often nor decreased the length of their sleep bouts, females spent a greater proportion of the night awake. Our study provides the first direct proof that light pollution has a significant impact on sleep in free-living animals, in particular in the morning, and highlights a mechanism for potential effects of light pollution on fitness.

Our natural environment is dramatically altered by increasing urbanization. One of its consequences is light pollution, which is defined as the alteration of natural light levels due to the introduction of artificial light at night. The rapid increase of artificial light at night, expansion of lit areas and increased light intensity, result in a loss of darkness with largely unknown consequences for biodiversity, ecosystems and ecological and evolutionary processes^1^,^2^. Light has a strong biological relevance for the daily and annual rhythms of life, given its periodic changes and/or seasonal fluctuations^3^,^4^,^5^, and there is accumulating evidence that light at night is not as harmless as previously thought. Laboratory studies found major disruptive effects from artificial light on a wide range of behavioural aspects such as reproduction, foraging, sleep and migration^6^,^7^,^8^,^9^,^10^,^11^,^12^,^13^. In addition, they also reported physiological effects including alterations in immune response^8^, cortisol levels^9^, melatonin levels^10^,^14^,^15^, testosterone levels^16^ and glucose metabolism^17^,^18^. There are, however, hardly any field studies on free-living animals and experimental manipulations of light conditions are almost entirely lacking (but see^19^,^20^,^21^).

Artificial light has a wide range of behavioural effects in birds. A study on black-tailed godwits (*Limosa limosa*) showed that they preferred to breed far away from artificial street light^22^. Light at night can also disorient and attract migratory birds, drawing them towards brightly lit objects such as offshore platforms^23^. Light pollution also attracts seabird fledglings which causes high mortality^13^,^24^. In several songbird species, including the great tit (*Parus major)*, it was shown that artificial light advanced the onset of activity and/ or dawn song^20^,^25^,^26^,^27^,^28^,^29^. As artificial light at night affects activity patterns of birds it is reasonable to assume that it also affects sleep behaviour. Blue tits (*Cyanistes caeruleus*) have been shown to adjust their awakening time according to local light conditions^30^. Hence, light pollution may cause animals to wake up earlier and potentially sleep less or, as cessation of activity can be delayed^31^, also fall asleep later. In contrast to laboratory studies (e.g.^32^), whether and how artificial light affects sleep behaviour in free-living birds has not yet been studied.

However, sleep is an important animal behaviour widespread across the animal kingdom^33^,^34^. There is clear evidence in many species that sleep allows animals to recover from daily stress^35^,^36^ and that sleep deprivation has major negative effects^33^,^34^. Sleep is common in bird species^37^,^38^ where it may not only serve to consolidate memory but also to conserve energy^39^,^40^,^41^. White-crowned sparrows (*Zonotrichia leucophrys gambelii*) can reduce sleep during migration without negative effects, however, outside the migratory season loss of sleep reduced cognitive functioning^32^. The few studies that have been carried out to study effects of natural or experimentally induced variation in sleep on fitness have produced mixed results until now. In the blue tit males that sleep longer are more likely to sire extra-pair offspring but otherwise there was no strong effect of variation in sleep behaviour on fitness^42^. Pectoral sandpipers (*Calidris melanotos)*, which breed in the arctic, can almost completely eliminate sleep without negative effects during the breeding season and males that sleep less sire more offspring^43^. There is also indirect and partial evidence from studies on activity patterns which suggest that variation in sleep might affect some aspects of fitness although much remains unknown. In blue tits animals that had an earlier dawn song (because of light pollution), suggesting that they slept less, had an advanced laying date and increased male extra-pair paternity^26^. Male great tits who had their activity experimentally delayed in the morning (through melatonin implementation, suggesting that they slept longer), had their fitness reduced through an increased risk of cuckoldry^44^. However, negative effects have also been reported. A case report on zebra finches (*Taeniopygia guttata)* suggests that sleep deprivation from exposure to continuous light led to increased mortality^45^. Although effects of sleep loss and disruption on fitness are largely unclear, it is an important first step to evaluate whether and how artificial light at night affects species in the wild.

Here we studied for the first time the impact of artificial light on sleep in free-living animals by quantifying its effects on sleep behaviour of great tits during the pre-breeding season. We experimentally provided male and female great tits, sleeping in nest-boxes, with artificial light to investigate the change in sleep behaviour compared to the natural dark situation. Individual sleep behaviour was, therefore, observed over two subsequent nights. We used a within-subject design in which the treatment group was provided with artificial light during the second night, the first night being used as a control. As an additional control we observed birds that slept in a natural dark situation during both nights.

## Methods

### Study area and general procedures

We collected data between February 17 and March 4 2014 in a resident suburban nest-box population of great tits in the surroundings of Wilrijk, Belgium (51°9’44”N, 4°24’15”E). Nest-boxes were put up in 1997, and this free-living population has been continuously monitored since then^46^. Great tits were caught inside nest-boxes during winter and breeding seasons after which they were sexed and ringed. Since 2012, all adults have been provided with a ring containing a passive integrated transponder (PIT) tag. This enables the individual detection of birds sleeping in nest-boxes without physically disturbing the birds.

### Experimental procedure

A paired design was used in which sleep behaviour was observed over two subsequent nights in a control (dark) treatment and a light treatment (Table S1). In the control group birds were observed over two nights sleeping in a naturally dark situation, while birds in the light group slept without a light turned on the first night and with a light turned on (see below) during the second night.

Observations of sleep behaviour in the control and light group were always performed simultaneously during one recording session (of two consecutive nights) with a total of six sessions. Paired data were obtained from nine individuals (three males and six females) in the control group and of 18 individuals (eleven males and seven females) in the light group. We expected minor differences in sleep behaviour between nights in the control group and therefore recorded fewer individuals in this group, compared to the light group.

### Recording of sleep behaviour and light treatment

Prior to the night of the first recording, all nest-boxes were checked during the night (at least one hour after sunset) for presence of a sleeping great tit by moving a handheld transponder reader (GR-250 RFID Reader, Trovan, Aalten, Netherlands) around the outside of the nest-box. Nest-boxes in which great tits had been sleeping were used in the experiment. During the experiment, nest-boxes were also checked every night with the transponder reader to ensure that the same individual slept in the nest-box on both nights. Infrared sensitive cameras (Pakatak PAK-MIR5, Essex, UK) were installed under the nest-box roof lid at least two hours before sunset and removed, at the earliest, two hours after sunrise the next morning (recordings started after installation). Ten infrared LED lights (which are invisible for great tits^47^) around the objective served as a light source for the camera.

Simultaneously with the video camera, we placed in each nest-box a small LED light (15 mm × 5 mm, taken from a RANEX 6000.217 LED headlight, Gilze, Netherlands) above the nest-box entrance hole on the inside, pointing downwards. These LEDs were standardized to produce 1.6 lux on the bottom of the nest-box as measured with an ISO-Tech ILM 1335 light meter (Corby, UK). In light polluted areas, birds are exposed to similar and higher light intensities, especially outside of nest-boxes or cavities^16^,^48^. In our population, those nest-boxes which are located near street lights, experience light intensities of more than 8 lux at the front of the nest-box opening. We chose white LED light because there is now a shift towards energy efficient broad spectrum light sources such as LED^49^,^50^.

We made recordings of the control group on two consecutive nights with a turned off LED light inside the nest-box. The first night that sleep behaviour was recorded in the light group, a LED light was present in the nest-box but turned off, thus birds slept in their normal dark situation similar to the control group. On the subsequent night the LED light was turned on at least two hours before sunset until at least two hours after sunrise the next morning. Thus, the light was turned on several hours before the birds entered the nest-box to go to sleep.

### Sleep parameters

Sleep of great tits was quantified in detail using 10 parameters: (1) entry time, (2) sleep onset, (3) evening latency, (4) awakening time, (5) leaving time, (6) morning latency, (7) sleep amount, (8) sleep proportion, (9) frequency of sleep bouts and (10) sleep bout length. We focused on these parameters as most have been used previously to study sleep behaviour in the closely related blue tit and have been associated with fitness related traits^30^,^42^.

We followed the definition of sleep parameters as described in Steinmeyer *et al.*^30^. In short, a bird was considered to be sleeping when it showed the classical sleep position (beak pointing backwards and tucked under the scapulars). Whether a bird was asleep or awake was usually easily distinguished. Only rarely was this distinction more difficult when individuals would occasionally sit quietly for some time with their head pointing forwards or not completely tucked under the scapular. These periods were defined as awake periods also because often they were followed by tucking the head under the shoulder. We define entry time and leaving time as the time when the bird entered or respectively left the nest-box. Sleep onset was defined as the first time a sleep bout of minimum 30 seconds had started. The time between entry time and sleep onset was defined as evening latency. Awakening time was defined as the last time the bird was asleep for at least 10 seconds. The sum of all sleep bouts was defined as sleep amount. We calculated sleep proportion as sleep amount divided by the total time spent inside the nest-box. The number of sleep bouts was calculated per hour as frequency of sleep bouts. All birds remained in the nest-box for the duration of the night after they had entered it in the evening. Some birds sat on the nest-box entrance hole several times before leaving in the morning, but only the moment when the bird had completely left the nest-box was used as leaving time. The time between awakening and leaving time was defined as morning latency.

In addition to the sleep parameters, we recorded activity during morning latency. During morning latency, the total time a bird spent on the nest-box entrance hole was used as “time on entrance” and the number of times it sat on the nest-box entrance hole was counted and used as “number of times on entrance”.

### Data analysis

Entry time, sleep onset, awakening time and leaving time were all converted to times relative to sunset or sunrise (reference data from Antwerp were used). For all statistical analyses we used R 3.0.2^51^.

We performed separate linear mixed effects analyses with the different sleep parameters as response variables (using the lme4 package^52^). As fixed effects, we entered treatment, date (Julian day), sex and night as well as the interactions between them (with the exception of interactions with date to avoid overfitting the model). Sex, as well as date, may influence sleep behaviour^30^ and were, therefore, entered in the model. As random effect, we entered bird identity nested in (recording) session to control for the repeated measures.

Where applicable, pairwise comparisons (using the multcomp package^53^) were used for post-hoc analyses, which provided t-values. Results are presented as marginal means with one standard error from the mean (S.E.; unless stated otherwise).

This study was approved by the ethical committee of the University of Antwerp (ID number 2011-31) and performed in accordance with Belgian and Flemish laws. The Belgian Royal Institute for Natural Sciences (Koninklijk Belgisch Instituut voor Natuurwetenschappen) provided ringing licences for authors and technical personnel.

## Results

In addition to the eighteen birds that we observed over two nights in the light group, there were nine birds who slept in a dark nest-box the first night but did not enter the nest-box during the second evening/ night when the LED light was on (these nine observations were excluded from further analyses). The proportion of birds not entering the nest-box the second evening was significantly higher in the light group compared to the control group (none of the nine birds; Fisher Exact Test, *P* = 0.026).

Male birds (*N* = 14) entered the nest-box later compared to female birds (*N* = 13) and their sleep amount was also reduced because of a later sleep onset. Other sleep parameters did not differ between sexes (see Table S2 for details).

### Effects of artificial light on sleep

**Figure 1:**
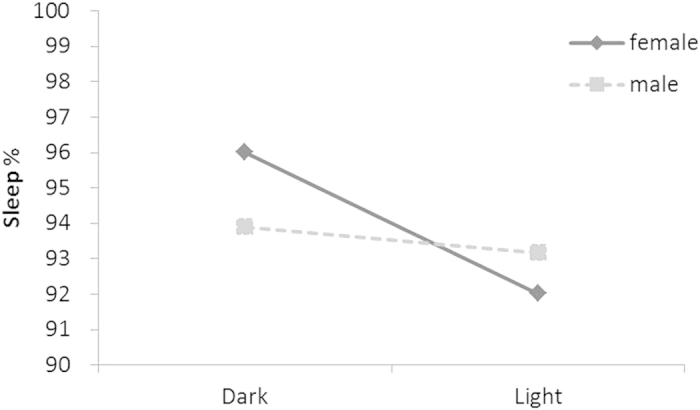
Effect of artificial light on the percentage of time spent asleep in the nest-box. Effects are shown for females (solid line) and males (dotted line) of the treatment group that first slept in a natural dark situation (on night 1) and subsequently with an artificial light turned on (during night 2). The difference was significant for females (*Z* = 8.65, *P* < 0.001), but not for males. P-value is obtained from a GLMM with bird identity nested in recording session (data were collected during six sessions; see Methods) as random factor to correct for repeated measurements. Mean and S.E. are shown (obtained from raw data).

While several aspects of sleep behaviour differed between sexes, the three way-interaction between sex, treatment and night was not significant for all but one sleep parameter, sleep proportion (*N* = 27*, χ*^2^_1_ = 13.123, *P* = <0.001; Table S2). The proportion of time spent sleeping in a lit nest-box was reduced for females (by about 4%; *N* = 27*, χ*^2^_1_ = 62.536, *P* = <0.001) but not for males (*N* = 27*, χ*^2^_1_ = 2.774, *P* = 0.096; Fig. 1 and Table S2).

**Figure 2:**
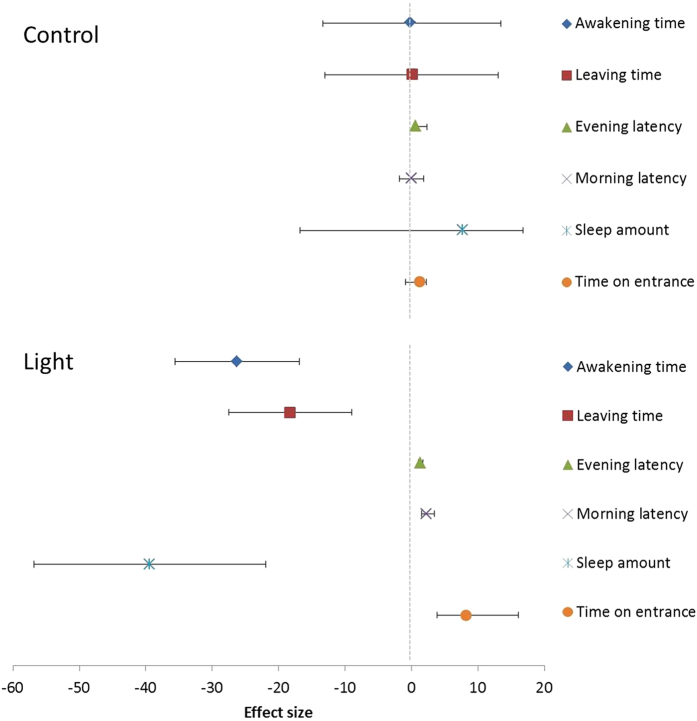
Effect of artificial light at night on sleep parameters. Shown are effect sizes and 95% confidence intervals of the contrast between the first and second night of sleep behaviour. Only sleep parameters that were significantly affected by artificial light are shown, with the top panel showing the effect sizes in the control group (sleeping in a natural dark situation on both nights), and the lower panel showing the effect sizes in the treatment group. Effect sizes are given in minutes, except for ‘time on entrance’ which is given in seconds, and are from a GLMM with bird identity nested in recording session (data were collected during six sessions; see Methods) as random factor to correct for repeated measurements.

Artificial light significantly affected most sleep parameters as indicated by the significant night*treatment interactions (Fig. 2, Tables S2 and S3). Exposed to the influence of artificial light, great tits woke up half an hour earlier (−26.3 min ± 4.5, *N* = 27*, t* = 5.79, *P* < 0.001) and left the nest-box 20 minutes earlier (−18.3 min ± 4.6, *N* = 27*, t* = 3.96, *P* < 0.001). Total sleep amount was reduced by almost three quarters of an hour (−39.4 min ± 8.6, *N* = 27*, t* = −4.56, *P* < 0.001) which amounts to a reduction of more than 5%. There was a small increase of several minutes in evening and morning latency (respectively 1.4 min ± 0.1, *N* = 27*, t* = −2.15, *P* = 0.04 and 2.2 min ± 0.2, *N* = 27*, t* = 3.91, *P* < 0.001). There was however no effect on sleep bout length or frequency (respectively: *χ*^2^_1_ = 0.512, *P* = 0.474 and *χ*^2^_1_ = 0.780, *P* = 0.377).

### Effects of artificial light on behaviour in the nest-box after awakening

Besides the effect of artificial light on morning latency, it significantly affected activity in the nest-box after awakening (see Fig. 2, Tables S2 and S3). When exposed to artificial light, birds went significantly more often to the nest-box entrance (1.5 ± 0.4, *Z* = 3.80, P < 0.001) and spent more time on it before leaving the nest-box (8.2 sec ± 0.4, t = −5.76, P < 0.001) compared to birds in the dark.

## Discussion

Previous studies reported that artificial light significantly affected activity patterns and the onset of dawn chorus in songbirds^20^,^25^,^26^,^27^,^28^, making it likely that sleep would also be affected. To our knowledge, our study is the first to demonstrate experimentally that artificial light does indeed disrupt sleep behaviour in free-living animals. Light at night caused birds to wake up earlier and leave the nest-box earlier in the morning, and as a result sleep less. Although individuals did not wake up more often at night females, but not males, spent a greater proportion of the night awake. During the night, sleep bout length and frequency were unaffected. In the evening, there was no direct effect of artificial light on sleep as birds did not fall asleep or enter the nest-box later. Nonetheless there was a small but significant increase in the time spent between entering the nest-box and falling asleep. Great tits were also less likely to enter an artificially lit nest-box.

Artificial light affected sleep in particular in the morning, with more subtle effects in the evening and during the night. We found that great tits woke up and left the nest-box earlier as a consequence of artificial light, perhaps because they perceived it as if the sun had already risen^19^,^30^. Birds in artificially lit nest-boxes also went more often to the nest-box entrance and spent more time on it during morning latency, which could be interpreted as birds being confused by the artificial light as it did not match with the light levels outside. In urban areas, similar behaviour might be found where there are street lights which are turned on in the morning which could “confuse” the birds. Additionally, the birds spent more time between waking up and leaving the nest-box, as demonstrated by the small but consistent increase in morning latency.

The increase in evening latency suggests that animals took longer to fall asleep after they entered the artificially lit nest-box. Although we found no direct evidence that artificial light affects sleep onset, Da Silva *et al.* found that street lights can prolong activity^25^, which could thereby indirectly cause animals to fall asleep later. Although most studies found that light pollution causes songbirds to advance the onset of activity^25^,^26^,^27^,^28^ the effects on cessation of activity are inconsistent between studies and species (see^27^,^28^,^54^ and^25^,^31^,^55^). Da Silva *et al.* showed that great tits continue singing longer at dusk because of light pollution^25^. However an experiment on activity patterns of great tits did not find artificial light to cause a later cessation of activity or an earlier onset^19^. There are two reasons which could explain this discrepancy in the results found on cessation of activity between our study and those of Da Silva *et al.*^25^ and Titulaer *et al.*^19^. First, the methodologies that were used differed between the studies. We used an experimental approach using lights installed inside nest-boxes, while the study of Titulaer *et al.*^19^ used lights which were installed outside of the nest-box (white LED lights, 10 lux). This potentially reduced the light intensity that actually reached the birds while roosting inside the nest-box. Da Silva *et al.* used a correlational approach comparing light polluted areas against dark areas. Our lights did not illuminate a substantial part of the environment outside of the nest-box and hence did not allow animals to be active for an extended period, contrary to light pollution caused by street lights^25^. Second, our study was conducted before the peak of the breeding season while the study of Da Silva *et al.* was conducted before and during the breeding season and the study of Titulaer *et al.*^19^ used nests with 1–16 day old chicks, which may have influenced leaving time^20^.

A study on the relation between emergence time and extra-pair paternity showed that female blue tits emerged 20 minutes earlier when an artificial light (white LED), also placed inside the nest-box, was switched on one hour before sunrise^20^. Whether, except for emergence time, also sleep was affected was not studied. Although the light we used was switched on for the entire night, the effect on leaving time appears very similar, suggesting that the effect of artificial light on awakening and leaving time could depend mainly on light intensity directly before sunrise. We used a relatively low light level (compared to street lights) which did not affect sleep bout length or frequency (sleep quality). However, females significantly reduced the proportion of time spent asleep in the nest-box. Turning lights off during part of the night, e.g. from midnight to 05:00 (as an alternative lighting strategy) may, therefore, still produce profound negative effects on sleep^56^ also because we found most effects in the morning. Nonetheless it could mitigate part of the effects on sleep behaviour of birds and mitigate other effects on a large diversity of other organisms^48^,^56^.

In general, the effect of artificial light on sleep did not differ between sexes in our study. However, we did find that females, unlike males, reduced the proportion of time spent asleep while in an artificially lit nest-box. It is difficult to explain this small but consistent effect, although it is known that male and female blue tits differ in their sleeping behaviour^30^ and we also found that in a natural dark situation male great tits entered the nest-box later, fell asleep later and slept less than females. Such sex differences could perhaps explain why the effect of artificial light differed between sexes for this aspect of sleep.

This study was performed before the breeding season. Other differences in how artificial light affects males and females could perhaps be found during the breeding season. Given that great tits sleep proportionally less during the breeding season than before the breeding season, (between 48 and 74% during the breeding season^57^ and around 94% in our study) and that the difference in sleep between blue tit males and females is greater near the breeding season^30^, it is important to carry out additional experiments at different periods of the year. However, during the breeding season mainly females occupy the nest-boxes making it more difficult to collect data on male sleep behaviour in great tits.

Previous research on effects of artificial light on activity patterns and onset of dawn chorus in songbirds^20^,^25^,^26^,^27^,^28^ provided only some clues that artificial light may reduce sleep as the results may have been caused either by birds falling asleep later and/or waking up earlier. Our results show that artificial light did not cause individuals to enter the nest-box or fall asleep later and that the effect on evening latency, although highly significant, only amount to a few minutes. We can therefore conclude that the reduction in sleep amount of almost three quarter of an hour (more than 5% reduction compared to a natural dark situation) mainly results from animals waking up earlier and that most effects occur in the morning. Experimental birds did not only spend less time in the nest-box but they also slept less because of artificial light. Individuals in the light group did leave the nest-box earlier and the advancement of awakening time was even larger. Surprisingly, animals did not wake up more often at night, nor did they spend longer periods awake between sleep bouts, indicating that artificial light during the night did not disrupt these aspects of sleep once birds were asleep.

With this study we provide the first direct experimental proof that light pollution can have a significant impact on several aspects of sleep behaviour. These results point out to a mechanism through which light pollution may affect fitness^42^ which requires further investigation. In blue tits it was shown that light pollution advanced their dawn song and that males had more extra-pair paternity^26^. Advancement of dawn song may indicate that light pollution also affected sleep, which is then correlated with increased male extra-pair paternity. In a different study it was shown that for female blue tits, artificial light advanced emergence time but did not affect extra-pair paternity^20^. However, experimentally delayed awakening and leaving time (through administration of melatonin and not light pollution) increased the risk of cuckoldry in great tits^44^. Quite clearly, further research is needed to assess the costs and benefits of disruption of sleep by light pollution.

As this is the first study on the effects of artificial light on sleep behaviour in the wild, we used a cavity-nesting bird as a model species. Because it is possible to manipulate light conditions within a nest-box, they are ideal study species to study effects of light on sleep in the wild. Experimental manipulation of light conditions of open-nesting birds is much more difficult. We believe that our results offer a first indication of how artificial light affects sleep in free-living birds and that these results could also be relevant for other animals exposed to light pollution as they are exposed to similar and even higher light intensities^16^,^48^. However, we recognise that besides similarities there are also differences in sleep between mammals, birds and invertebrates^58^,^59^,^60^ as well as between different bird species (e.g.^42^,^43^). For instance, sleep could differ between species because of differences in exposure to predators^61^. Although sleep quantity was affected, we did not find any effect on sleep quality (measured behaviourally) during the pre-breeding season using a light intensity of 1.6 lux. However, there is now an urgent need to perform similar studies in different periods of the year, using different light intensities and to look at the effects of this sleep disruption on fitness. A proper assessment of short and long-term effects on fitness would require a larger sample size than we used here and perhaps also a longer period of light exposure.

Our experimental setup has great potential for further research to elucidate short term physiological effects as well as short- and long term fitness effects of artificial light in free-living birds. Using our experimental setup, the light regime could also be manipulated in terms of light intensity, duration and spectra (light colours) in future studies. This is necessary for crucial research on the effects of light pollution^48^ and can be used to investigate the effectiveness of management options to reduce consequences of artificial light at night^56^. Our experimental setup and approach can be used with other cavity-nesting species and at different times of the year (outside versus within the breeding season), which will undoubtedly yield new insights about the effects of light pollution under natural conditions.

## Additional Information

**How to cite this article**: Raap, T. *et al.* Light pollution disrupts sleep in free-living animals. *Sci. Rep.* **5**, 13557; doi: 10.1038/srep13557 (2015).

## Supplementary Material

Supplementary Information
