# Supplementary material for: Light pollution disrupts sleep in free-living animals
Source: Sci Rep. 2015 Sep 4;5:13557. doi: 10.1038/srep13557 (PMC4559670; doi:10.1038/srep13557)
Supplement: Supplementary Information [file srep13557-s1.pdf]

Supplementary material

Light pollution disrupts sleep in free-living animals

Thomas Raap[1]*

Rianne Pinxten[1,2]

Marcel Eens[1]

[1]Department of Biology, Ethology group, University of Antwerp, Universiteitsplein 1, B-2610 Wilrijk, Belgium

[2] Department of Instructional and Educational Sciences, Didactica research group, University of Antwerp, Prinsstraat 13, B-2000, Antwerp, Belgium

* Corresponding author: thomas.raap@uantwerpen.be

Methodology

Selection of nest-boxes

Nest-boxes with a maximum light intensity of 0.3 lux (range: 0.01 - 0.26 lux, average: 0.12 lux) at the entrance hole of the nest-box were selected for this experiment (light intensity inside nest-boxes: ± 0.01 lux). These nest-boxes were not under the direct influence of street lights but under a natural light regime. Light measurements were carried out from January 27 till January 31 2014 at least one hour after sunset with a light meter (ISO-Tech ILM 1335, Corby, UK; range 0.01 to 30000 lux) at the nest-box entrance hole. The light meter was placed vertically on the entrance and the maximum value of light intensity was obtained.

Statistical analysis

We analysed the relationship between sleep parameters using a Spearman rank correlation test (in the psych package[1]) on the sleep behaviour of the first night (dark control) of both treatment groups. To avoid pseudoreplication the second night of the control group was not used. Although we found significant correlations between some sleep parameters (sleep onset and entry time as well as sleep bout and sleep bout per hour; Table S4), we chose to analyse each parameter separately as artificial light could influence sleep parameters independently from each other. We followed Steinmeyer et al.[2] in testing all sleep parameters even though they can be correlated, as they found that they can be affected independently from each other (natural light affected awakening time but not leaving time).

Since each bird was tested more than once, and the birds are tested within sessions, we cannot assume independence between the observations in this dataset. This violates a key assumption of the classic ANOVA and regression analysis, that the error terms are independent. Also

non-parametric tests assume that all observations are independent.

Mixed models are a widely used technique to account for the non-independence between observations in a dataset, by including random effect terms into the regression equation. The significance of the independent variables (“fixed effects”) is hereby calculated, accounting for the non-independence of the observations within the same individual (or session)[3].

Evening and morning latency and time on entrance were log transformed to reduce right-sided skew. To test whether artificial light changed sleep behaviour from the first night compared to the second night, we tested the interaction night*treatment. Activity during morning latency (time on entrance and number of times on entrance) was analysed with a linear mixed effects analysis (see main text). A generalized linear mixed model with a Poisson error structure was used to analyse the effect of artificial light on “number of times on entrance”. We were mainly interested in the interactions sex*treatment*night and night*treatment to show the effect of artificial light on sleep and in the main effects sex and date as control variables.

Model selection was done by backward elimination of non-significant ($P > 0.05$) factors and interactions, starting with the highest order interaction. P-values were obtained by likelihood ratio tests of the full model against the reduced model.

Table S1. Experimental design. The number of individuals is given per treatment. A total of 54 observations from 27 individuals were obtained and used in the analysis of differences in sleeping behaviour.

**Treatment**	**Night (light off/ on)**		**Observations**	**Individuals total (*N*)**	**Male (*N*)**	**Female (*N*)**
	**1**	**2**				
Control	OFF	OFF	18	9	3	6
Light	OFF	ON	36	18	11	7
Total			54	27	14	13

Table S2. Effect of sex, date and artificial light (treatment) on sleep parameters. To correct for changes in day length, response variables were standardised to civil sunset (entry time, sleep onset) or sunrise (awakening time and leaving time). GLMM models were used with bird identity nested in recording session (data were collected during six sessions; see Methods) as random factor to correct for repeated measurements. Significant P values are shown in bold, *N* = 27.

Sleep parameter	Sex[a]			Date[b]			Night*Treatment		Sex*Treatment *Night	
	Estimate ± SE	X^2	p-value	Estimate ± SE	X^2	p-value	X^2	p-value	X^2	p-value
Entry time	15.05 ± 3.96	12.389	**<0.001**	0.04 ± 0.68	0.004	0.950	1.055	0.304	1.368	0.242
Sleep onset	15.00 ± 3.94	12.757	**<0.001**	0.05 ± 0.69	0.006	0.937	2.418	0.120	1.104	0.293
Awakening time	-7.71 ± 4.36	3.1269	0.096	0.06 ± 0.70	0.006	0.941	9.949	**0.002**	0.900	0.353
Leaving time	-4.94 ± 3.88	1.755	0.185	0.18 ± 0.38	0.247	0.619	5.429	**0.020**	1.426	0.232
Evening latency[c]	-0.03 ± 0.13	0.086	0.770	0.01 ± 0.01	0.596	0.440	10.891	**0.001**	0.523	0.470
Morning latency[c]	0.12 ± 0.22	0.402	0.526	0.02 ± 0.06	0.242	0.623	5.153	**0.023**	1.425	0.233
Time on entrance[c]	0.05 ± 0.38	0.072	0.789	0.08 ± 0.11	0.860	0.354	8.633	**0.003**	1.337	0.248
Nr times on entrance	-0.12 ± 0.26	0.251	0.616	0.01 ± 0.04	0.135	**0.714**	13.814	**<0.001**	0.043	0.836
Sleep amount	-4.01 ± 8.22	4.294	**0.038**	-3.17 ± 1.19	7.047	**0.008**	9.669	**0.002**	1.553	0.213
Sleep bout (sec)	-42.06 ± 61.73	0.512	0.474	11.06 ± 6.05	3.192	0.074	2.479	0.115	0.261	0.609
Sleep bout/h	0.39 ± 0.49	0.780	0.377	-0.09 ± 0.06	2.981	0.084	1.835	0.176	1.4429	0.230
Sleep %	-	-	-	-0.01 ± 0.01	0.827	0.363	-	-	13.123	**<0.001**
Sleep % male	-	-	-	-	-	-	2.774	0.096	-	-
Sleep % female	-	-	-	-	-	-	62.536	**<0.001**	-	-

[a] Effects of sex (females-males) were estimated as differences in means.

[b] The effects of date are given as slopes.

[c] Values were log transformed to reduce skew.

Table S3. Descriptive statistics of sleep in great tits under natural dark conditions and the effect of artificial light on them. Entry time and sleep onset are standardised to civil sunset and awakening, and leaving time are standardised to sunrise. SD is standard deviation, CI is 95% confidence interval. See also table S2 and figure 2.

	Natural conditions				Effect of artificial light		
Sleep parameter (min)[#]	Average	Min	Max	SD	Estimate	CI low	CI high
Entry time	-4.9	-33	22	15.2			
Sleep onset	-0.2	-30	24	15.1			
Awakening time	-26	-43	-10	8.8	-26.3	-16.9	-35.6
Leaving time	-21.8	-34	-9	7.4	-18.3	-9.0	-27.6
Evening latency	4.7	2	13	2.5	1.3	1.7	1.0
Morning latency	4.7	0	11	3.5	2.2	3.5	1.5
Time on entrance*	47.3	2	261	63.9	8.2	16.0	3.8
Nr times on entrance	1.8	1	5	1.0			
Sleep amount	731.5	694	807	29.9	-39.4	-22.0	-56.8
Sleep bout*	678.5	317	1040	183.4			
Sleep bout/h	5.4	3.3	10.1	1.6			
Sleep proportion	94%	89%	98%	2%			

[#] Unless stated otherwise

* Values in seconds.

Table S4. Correlation coefficients describing the relationship between sleep parameters. Spearman rank correlation tests with P-values adjusted for multiple testing (Holm method) were used on the averaged data from control nights. Significant correlations ($P < 0.05$) are shown in bold, trends ($P < 0.10$) are underlined, other correlations are not significant, $N = 27$.

Sleep parameters	Entry time	Sleep onset	Evening latency	Awakening time	Leaving time	Morning latency	Sleep amount	Sleep %	Sleep bout/h
Sleep onset	**0.99**								
Evening latency	-0.01	0.06							
Awakening time	-0.27	-0.26	-0.05						
Leaving time	-0.59	*-0.59*	-0.03	0.60					
Morning latency	0.34	0.35	0.31	0.00	-0.26				
Sleep amount	-0.58	-0.57	-0.06	0.13	0.53	-0.40			
Sleep %	0.14	0.12	-0.45	-0.02	0.09	-0.44	0.38		
Sleep bout/h	0.23	0.21	-0.09	-0.12	-0.17	0.12	-0.39	-0.19	
Sleep bout (sec)	-0.24	-0.22	0.05	0.13	0.22	-0.15	0.41	0.25	**-0.99**

References

1 psych: Procedures for Psychological, Psychometric, and Personality Research v. version 1.3.1 (Northwestern University, Evanston, Illinois, USA, 2014).

2 Steinmeyer, C., Schielzeth, H., Mueller, J. C. & Kempenaers, B. Variation in sleep behaviour in free-living blue tits, Cyanistes caeruleus: effects of sex, age and environment. *Anim. Behav.* **80**, 853-864, doi:10.1016/j.anbehav.2010.08.005 (2010).

3 Fitzmaurice, G., Laird, N. & Ware, J. *Applied longitudinal analysis*. (Wiley, 2004).
